# Herd-Level Risk Factors for Swine Influenza (H1N1) Seropositivity in West Java and Banten Provinces of Indonesia (2016–2017)

**DOI:** 10.3389/fvets.2020.544279

**Published:** 2020-11-11

**Authors:** Nurhayati, Hendra Wibawa, Trian Mahawan, Farida Camallia Zenal, Luuk Schoonman, Caitlin Nicole Pfeiffer, Mark Stevenson, Veerasak Punyapornwithaya

**Affiliations:** ^1^Faculty of Veterinary Medicine, Chiang Mai University, Chiang Mai, Thailand; ^2^Directorate of Animal Health, Directorate General Livestock and Animal Health Services, Ministry of Agriculture, Jakarta, Indonesia; ^3^Disease Investigation Center Wates, Yogyakarta, Indonesia; ^4^Disease Investigation Center Subang, West Java, Indonesia; ^5^Food and Agriculture Organization of the United Nations, Emergency Centre for Transboundary Animal Diseases, Jakarta, Indonesia; ^6^Faculty of Veterinary and Agricultural Sciences, The University of Melbourne, Parkville, VIC, Australia; ^7^Veterinary Public Health and Food Safety Centre for Asia Pacific, Faculty of Veterinary Medicine, Chiang Mai University, Chiang Mai, Thailand

**Keywords:** swine influenza virus, risk factors, seroprevalence, Indonesia, swine

## Abstract

Swine could play a role as a “mixing vessel” for avian and human influenza viruses and should, therefore, be thought of playing an intermediate role in the emergence of pandemic influenza strains. The aim of this study was to identify risk factors for Swine influenza virus (SIV) seropositivity at the farm level in West Java and Banten provinces, Indonesia. A total of 649 blood samples were collected from 175 pig farms, and at the time of sampling, a questionnaire about routine herd management was administered to participant herd managers. Swine influenza virus serological status for each of the sampled pigs was tested using the IDEXX ELISA-test (Maine, US). The apparent herd-level prevalence of SIV seropositivity was expressed as a true herd-level prevalence using the Rogan and Gladen method, modified to account for low and high prevalence herds using a Markov chain Monte Carlo Bayesian approach. The association between herd-level characteristics and SIV seropositivity status was assessed using binary logistic regression. The true prevalence of SIV seropositivity was 26% (95% CI = 20–33). The presence of animals apart from pigs on farm (odds ratio, OR = 2.51, 95% CI = 1.0–6.0), keeping breeding sows for <2 years (OR = 5.9, 95% Cl = 1.8–20), being <1 km from a poultry farm (OR = 2.4, 95% Cl = 1.0–5.7), and purchasing pigs only through pig collectors (OR = 11, 95% CI = 4.3–29) increased the risk of a herd being seropositive to SIV. Our results show that biosecurity to limit the introduction of SIV should be enhanced on farms located in areas of high pig and poultry farm density. While the role that pig collectors play in the transmission of SIV warrants further investigation, swine producers in West Java and Banten should be made aware of the enhanced risk of SIV associated with purchasing of replacements from collectors.

## Introduction

Swine influenza virus (SIV) infection is an acute and contagious respiratory disease of pigs (1, 2) that causes economic loss in commercial piggeries due to high morbidity (3, 4). In addition, the presence of swine influenza raises public health concerns because pigs can be infected by other pigs, poultry, or human influenza viruses at the same time, and this could potentially generate a novel pandemic strain (4–6). Although people who work in close contact with pigs have been reported to have an increased seroprevalence to SIV (7, 8), the incidence of SIV among humans has rarely been investigated.

Swine influenza is caused by an influenza type A virus that belongs to the Orthomyxoviridae family. Influenza type A viruses can be categorized based on their hemagglutinin and neuraminidase proteins. In pigs, influenza type A viruses are often detected as H1N1, H1N2, and H3N2 sub-types (9). One relatively stable subtype is H1N1, the etiologic agent responsible for most swine influenza outbreaks until the mid-1990s and historically associated with classical swine flu strains. The primary route of virus transmission is pig-to-pig contact, with the virus entering the body *via* the nasopharyngeal route, most probably through nose-to-nose contact or following direct contact with mucus.

Swine flu outbreaks have been reported in several parts of the world. In America, swine flu was first reported in the north and mid-west of the United States in 1918. However, the virus could only be isolated in pigs in 1930 (10). The classical SIV in Europe was first isolated during an outbreak that occurred in northern Italy in 1976 (10). Based on surveillance conducted in 2006 and 2007, swine flu has caused acute respiratory distress in pigs in Belgium, England, Italy, France, and Spain (11). Swine flu infections in humans caused by H1N1 and H3N2 subtypes were reported in Italy in 1993 (12). A serologic surveillance in Japan indicates that H1N1 subtype influenza infection has occurred in Asia since 1977 (13). In Southern China, isolation of the swine influenza H1N1 virus subtype was carried out in 1993 (14). In Indonesia, H1N1 influenza (the pandemic strain of 2009) was reported in April 2009 (15). The virus was detected at pig slaughterhouses in the province of Jakarta and on pig farms in Bulan island, in the province of Riau Islands, in 2009 (15). The 2009 epidemic of SIV in Indonesia was responsible for 1,005 confirmed cases and five deaths (15).

Information on SIV-related risk factors in pig farms is limited, although some studies have reported on some risk factors. A study in England indicate that keeping pigs indoor, high density of pigs per water space, and younger pig age (16) are potential risk factors for SIV infection in pigs. The existence of a pen partition between pens, uncontrolled entrance to the farm (17), and history of a respiratory illness of pigs (18) have been identified as risk factors for influenza seropositivity of pig farms in Spain and China. In addition, the size of the farm and the presence of other animals have been reported as risk factors for the spread of SIV among pigs in pig farms in Malaysia, the neighboring country of Indonesia (19).

Indonesia has reported large numbers of outbreaks of HPAI H5N1 in poultry since 2003 (20). While numerous epidemiological studies of HPAI H5N1 in poultry in Indonesia have been published (21–24), studies on the epidemiology of SIV are limited. We could locate only one field study of SIV that concentrated on estimations of seroprevalence (25). With this background, our aims were to describe the prevalence of SIV seropositivity among commercial swine herds in Java, Indonesia, and to identify the risk factors for SIV seropositivity. Better knowledge of the risk factors for SIV provides insight into farm-level and herd management characteristics that increase the risk of the disease.

## Materials and Methods

### Study Area

The study areas were Banten and West Java provinces, bordering Jakarta (the capital city of Indonesia) where the highest number of human cases due to highly pathogenic avian influenza H5N1 (HPAI) were reported during 2005–2017 (26). During the HPAI outbreak, most of the poultry farms in both West Java and Banten were infected by the disease (27). Importantly, the two provinces supply pork meat to Jakarta. The number of pig farms in Banten province was 135 (4,823 pigs), with a density of 0.49 pigs/km^2^, while the number of farms in the province of West Java was 310 (7,055 pigs), with a density of 0.199 pigs/km^2^ (28). Apart from having pig farms, the provinces of West Java and Banten also have poultry farms, either commercial poultry or backyard type. The transmission of zoonotic SIV to humans is an important public health concern for the study areas.

### Sample Size

Sample size determination was performed using ProMESA software, version 1.62 (EpiCenter, Massey University, New Zealand), aiming to detect the presence of SIV in a pig population based on a two-stage sampling design. The two-stage sampling was determined by calculating independently the number of herds from which the individuals will be sampled and the number of individuals per herd to include in the sample. Several parameters were defined for sample size calculation, including the total number of pig farms (*n* = 445), average number of pigs per farm (*n* = 30), minimum expected prevalence of positive herds (1.5%), and minimum expected prevalence of positive animals (50%). The output indicated that this study required at least 166 pig farms and at least four animals per farm to be sampled. On each farm, young and adult pigs were randomly selected for blood collection.

### Study Design

**Figure 1:**
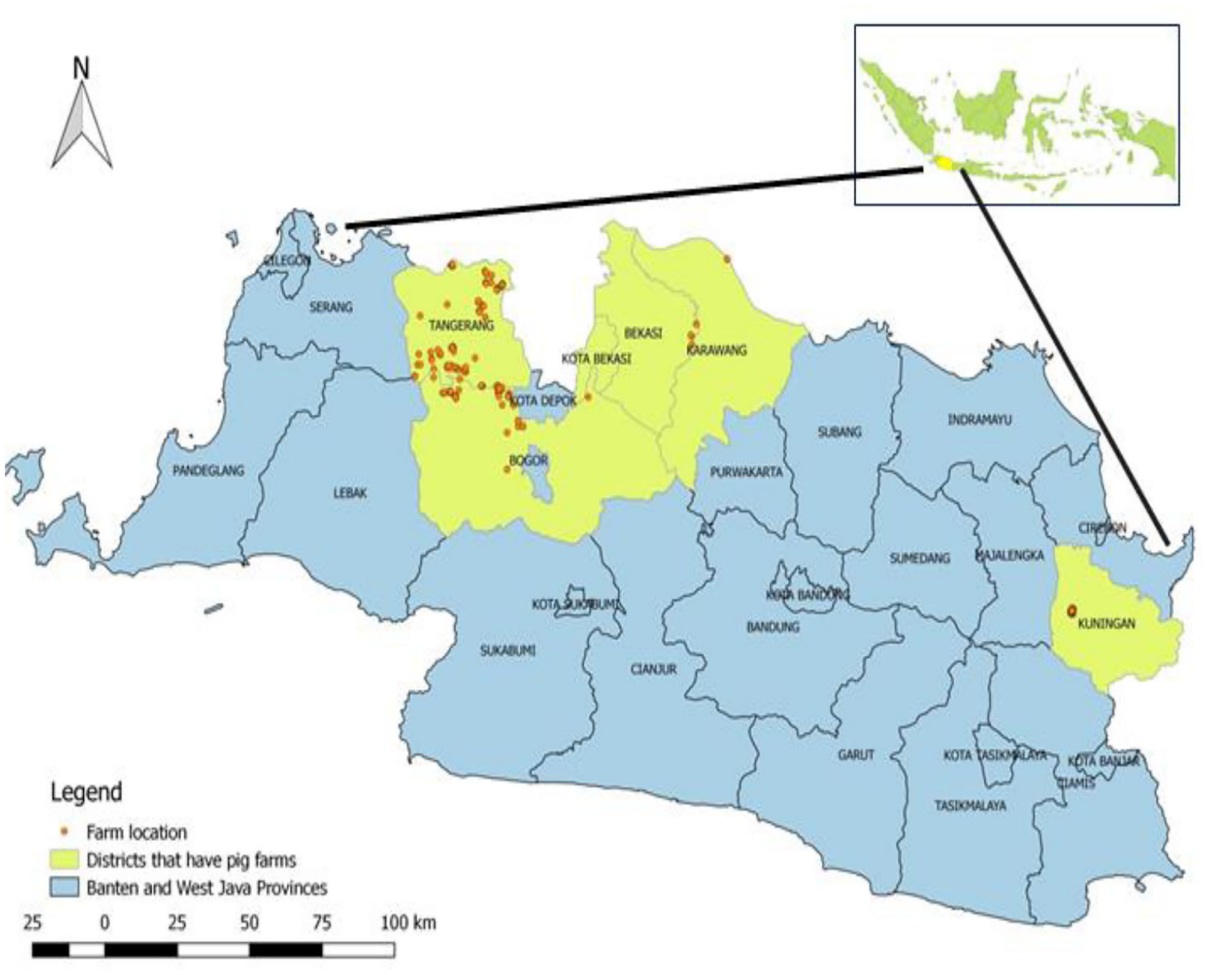
Geographical map of the Republic of Indonesia and the study area including Banten province (Tangerang City, Tangerang) and West Java (Bekasi, Bogor, Karawang, and Kuningan). Districts where enquiries were conducted are yellow in color, and farms are represented as red, round dots.

This was a cross-sectional study conducted as part of a national pig disease surveillance program carried out by the Disease Investigation Center Subang within the Directorate General of Livestock and Animal Health Services of the Ministry of Agriculture, Indonesia. The study was carried out from February 2016 to November 2017 in two districts in Banten province (Tangerang, Tangerang City) and four districts in West Java province (Bogor, Bekasi, Karawang, and Kuningan; Figure 1). The total number of swine farms in the study area was 445, of which 175 were selected for sampling.

On each farm, at least three animals (young and adult) were selected at random for blood collection. The number of pigs to be sampled was chosen to provide 95% confidence that at least one seropositive pig would be detected if the within-herd prevalence of SIV seropositivity was 50%. Samples were collected by jugular vein puncture using plain evacuated tubes (Vacuette, Dutscher SAS, Brumath, France). Sera were obtained by centrifugation for 10 min at 3,500 × *g* and stored at −20°C until testing.

### Farm Data

At the time the farms were visited for sampling, a face-to-face interview with the herd manager was carried out using a standardized questionnaire. To ensure consistency in the way responses to questions were recorded, district officers who carried out the sampling and administered the questionnaire were trained on how to conduct an interview, clarify questions, and conduct operational procedures. All the herd managers that consented to having their pigs sampled agreed to take part in answering the questionnaire.

The questionnaire solicited details about general herd information, health management, and sources of pigs. The general herd information section of the questionnaire recorded details of farm location (the longitude and the latitude of the main farm shed were recorded by the district officer administering the questionnaire using a global positioning system), the type of herd, the reason for keeping pigs, the type of business (fattening/breeding), the length of time pigs were raised, herd management, use of personal protective equipment, details of biosecurity and farm access, and distance to the nearest residential area. Farm management details included the type of management system (intensive, free range/extensive), the type of buildings and cages used, the distance from the herd manager's home to the farm, the presence or the absence of other animals on the farm, the distance of the farm to the nearest commercial poultry farm, the type of feeders and waterers in use, the presence or the absence of slaughter facilities within the farm, the number of animals of different age classes present, and information relating to waste management. The health management section of the questionnaire included questions about the presence or the absence of a vaccination program, the presence or the absence of a worm control program, and information relating to the frequency of disease events during the 3-month period immediately prior to the administration of the questionnaire. Questions about the source of pigs included the origin of replacement gilts and boars, the destination of pigs that were sold, and the method of transport of sold pigs. Questionnaire data were entered into a relational database software for analysis.

### Serological Data

**Figure 2:**
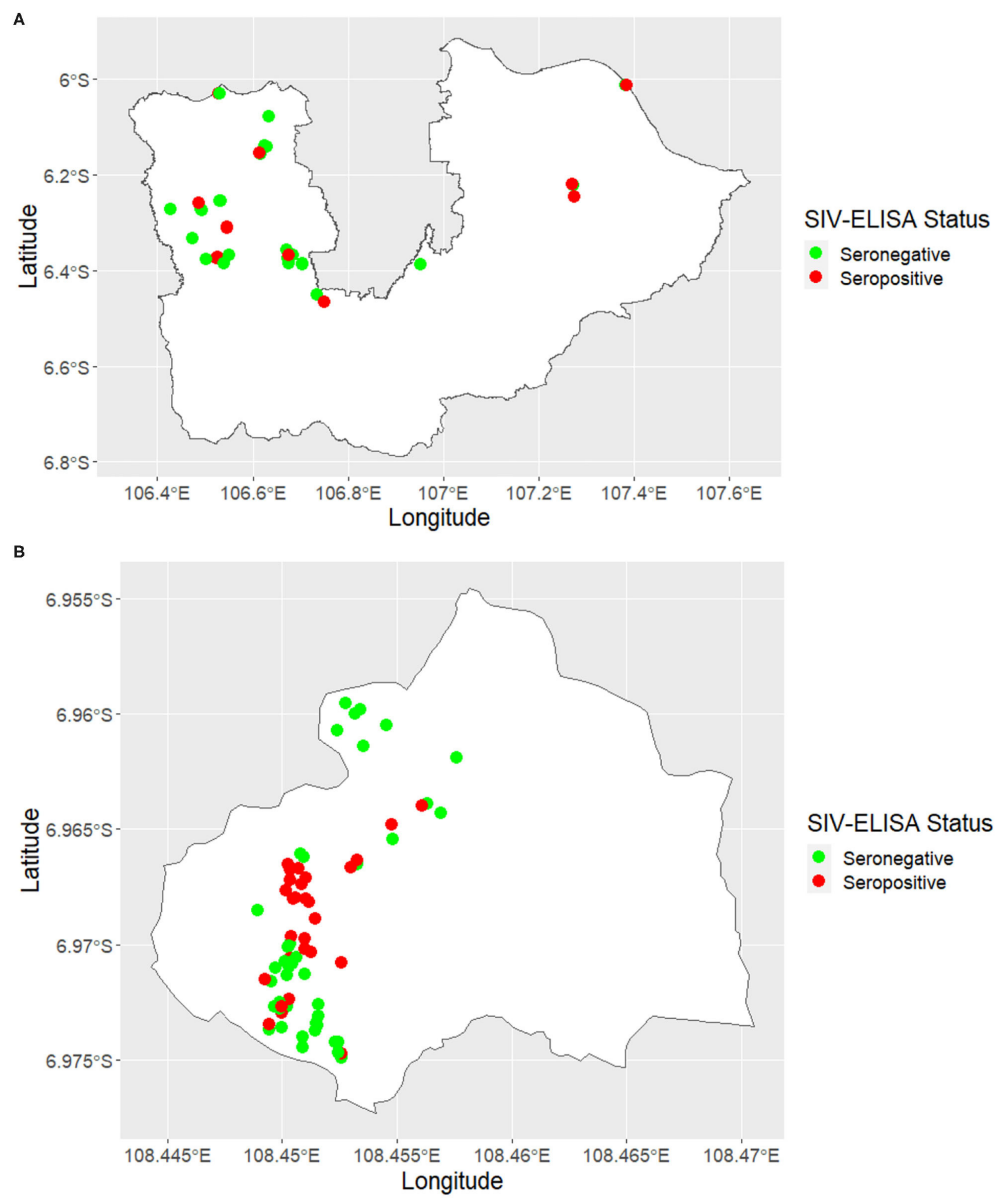
Maps showing the location of swine influenza farm status classified by serological-test results in **(A)** Tangerang City, Tangerang, Bogor, Bekasi, and Karawang and **(B)** Kuningan.

A total of 649 blood samples were collected from 175 pig farms in six districts of West Java and Banten, including Tangerang (*n* = 22), Tangerang City (*n* = 6), Bogor (*n* = 30), Bekasi (*n* = 4), Karawang (*n* = 3), and Kuningan (*n* = 110). The locations of the farms sampled within each of the six districts are shown in Figure 2.

The SIV serology status for each sampled pig was assessed using the ELISA-test for detection of influenza A nucleoprotein (NP)-specific antibodies using a commercial kit (IDEXX® influenza A Test Kit, Maine, USA). The presence or the absence of antibody to influenza A was determined using the sample to negative (S/N) ratio. According to the manufacturer's instructions, samples were identified as positive if the value of S/N was <0.5 and negative if S/N was ≥0.5. According to a previous report (29), the diagnostic sensitivity of the IDEXX influenza A is 86% (95% CI, 76–90%), and the diagnostic specificity is 79% (95% CI, 63–90%).

### Statistical Analyses

We report both the apparent and the true herd-level prevalence of SIV seropositivity using the IDEXX ELISA. The apparent herd-level prevalence (AP) of SIV seropositivity was defined as the number of IDEXX ELISA-positive pigs per herd divided by the total number of pigs tested. True herd-level prevalence (TP) estimates take into account the imperfect diagnostic sensitivity (Se) and specificity (Sp) of the IDEXX ELISA using the approach (30) and modified for the extreme (i.e., low or high) prevalence situation using Bayesian methods (31). If *x* equals the number of pigs testing positive using a diagnostic-test of sensitivity Se and specificity Sp and *n* equals the number of pigs tested, the distribution of the number of test-positive pigs equals:

$$\begin{array}{l} \text{x}|\left(TP,Se,Sp\right)\sim binomial\left(\text{n},AP\right)\\ whereAP=TP\;*\;Se+\left(1-TP\right)\;*\;\left(1-Sp\right) \end{array}$$

To estimate the true prevalence of SIV seropositivity in each herd, beta prior distributions for Se and Sp were used. For the IDEXX ELISA, we assumed that the mode of the diagnostic sensitivity was 0.86 and that we were 95% confident that the diagnostic sensitivity was >0.76 (29). Similarly, we assumed that the mode of specificity of the IDEXX ELISA was 0.79 and that we were 95% confident that diagnostic specificity was >0.63 (29).

Markov chain Monte Carlo (MCMC) methods were used to derive posterior estimates of the within-herd TP of SIV exposure using JAGS (32). Using JAGS, the MCMC sampler was run for 100,000 iterations and the first 5,000 “burn in” samples discarded. The posterior distribution of TP was obtained by running sufficient iterations to ensure that the Monte Carlo standard error of the posterior means was at least one order of magnitude smaller than their posterior standard deviation (33). Herds were classified as SIV-positive if the within-herd TP of SIV exposure was >0 and SIV-negative if otherwise.

The association between general herd information characteristics, health management characteristics, and sources of pigs and herd-level SIV seropositivity status (defined on the basis of the true herd-level prevalence of SIV seropositivity, described above) was quantified using binary logistic regression (34). Putative risk factors associated with the outcome of interest at a significance level of *p* ≤ 0.25 were selected for multivariable binary logistic regression modeling.

Pairs of putative explanatory variables that were associated with herd-level SIV status at *p* ≤ 0.05 were checked for multicolinearity using chi-square-test for categorical variables. In the presence of statistically significant collinearity (*p* ≤ 0.05), the variable considered to be the more biologically plausible risk factor for SIV was retained for multivariable logistic regression analysis.

A backward elimination process was used to select explanatory variables associated with herd SIV status. All putative explanatory variables that were associated with the outcome variable were entered into the model. Explanatory variables that were not significantly associated with herd SIV-seropositivity status were removed from the model one at a time, beginning with the least significant, until the estimated regression coefficients for all variables retained were significant at an α level of <0.05.

The final model's goodness-of-fit was evaluated using the Hosmer–Lemeshow-test, and the ability of the model to discriminate between SIV-seropositive and SIV-seronegative herds was assessed by constructing a receiver operating characteristic (ROC) curve. The area under the ROC curve, which ranges from zero to one, provided a measure of the model's ability to discriminate SIV-seropositive and SIV-seronegative herds. The greater the area under the ROC curve, the greater the model's discriminatory power (34).

## Results

A total of 175 farms were included in this study, with 147 of 175 (84%) of farms in West Java and 28 of 175 (16%) in Banten. The average number of pigs per farm in both provinces was 67.5 (median = 38, Q1 = 20, Q3 = 78.5), with most herd managers describing their enterprise type as non-commercial (155 of 175, 89%). In 170 of 175 (97%) farms, pigs were kept inside cages all day, while in the remainder of the herds, the pigs were kept in cages but could still have contact with other animals (e.g., birds, cats, or dogs). Most herd managers kept their sows for <2 years (138 of 175, 79%), and most herd managers kept animals such as dogs, cats, free-range chickens, and birds on farm (104 of 175, 60%). Seventy-one of 175 herd managers (41%) kept only pigs on farm. Ninety-one of 175 farms (52%) were located within the 1-km radius of commercial poultry farms, and 24% (42 of 175) of herd managers bought replacement pigs only from collectors, while the remaining 76% (133 of 175) bought replacement pigs from both other farmers and collectors.

In total, 649 serum samples were collected from 175 farms and tested for SIV H1N1, with 157 samples returning a positive result. The true herd-level prevalence of SIV seropositivity was 26 (95% CI, 20–33) herds per 100 herds at risk. SIV-seropositive farms were identified in all four districts (Bogor, Bekasi, Karawang, and Kuningan) of West Java and both two districts (Tangerang City and Tangerang) of Banten. Maps showing the location of SIV-seropositive and SIV-seronegative herds are shown in Figures 2A,B, respectively.

Our univariate analyses were carried out on 29 putative explanatory variables, with 16 of them associated with herd-level SIV seropositivity status at *p* <0.25 (Supplementary Table 1). For multivariable analysis, four risk factors increased the risk of a farm to being SIV-seropositive: keeping animals apart from pigs on farm, keeping sows for <2 years, being <1 km away from a poultry farm, and purchasing pigs only through pig collectors (Table 1).

**Table 1:** Herd-level risk factors H1N1 swine influenza seropositivity, West Java and Banten provinces, Indonesia, 2016–2017, from multivariable logistic regression model.

**Variable**	**OR (95% CI)**	***p*-value**
**Presence of other animal species on farm**		
Yes	2.5 (1.0–6.0)	0.03
No		
**Length of time sows were kept on farm**		
Less than 2 years	5.9 (1.8–20)	<0.01
More than 2 years		
**Distance to nearest poultry farm**		
Less than 1 km	2.4 (1.0–5.7)	0.03
More than 1 km		
**Replacement pigs purchased only from a collector**		
Yes	11 (4.3–29)	<0.01
No		

*Cox and Snell, R^2^ (p = 0.22); Hosmer–Lemeshow-test (p = 0.99)*.

Our model provided an acceptable ability to discriminate between SIV-seropositive and SIV-seronegative herds, with the area under the ROC curve equal to 0.78. The model's accuracy was moderate to good (accuracy = 0.80; 95% CI, 0.73–0.85). While the model's sensitivity was low (0.36; 95% CI, 0.23–0.52), its specificity was good (0.95; 95% CI, 0.89–0.98).

## Discussion

One in four of the herds included in this study was SIV-seropositive, and SIV-seronegative herds were identified in all the six districts included in the study area. Our results show that the prevalence of SIV exposure in swine herds in this part of Indonesia is relatively high and that the SIV-seropositive herds in Java were geographically dispersed.

The strength of the association between each of the explanatory variables and herd SIV seropositivity status was similar in both the univariable and the multivariable analyses, which implies that none of the explanatory variables included in the multivariable analysis was an important confounder. Our finding that the odds of a herd being SIV-seropositive increased if other animal species (such as cats, dogs, and/or poultry) were kept on farm is in broad agreement with those of other studies. A cross-sectional study in Malaysia in 2005 (19) found that the presence of pets on farm (e.g., cats) was associated with an increased risk of H1N1 and H3N2 infection in pigs. Other studies have shown that the presence of poultry on pig farms was associated with an increased risk of swine being seropositive for SIV (17, 25). Pigs are susceptible to influenza virus infection from poultry and other mammals. For this reason, introducing and keeping other animals (e.g., dogs, cats, and poultry) is not recommended.

Our study showed that the risk of SIV seropositivity was increased on those farms where breeding sows were kept for <2 years. Swine farmers in this area of Indonesia did not routinely practice an “all in, all out” farm management system, which means that there is a relatively constant turnover of breeding sows entering and exiting a farm enterprise at any point in time. A management system whereby sows are kept for a relatively short period of time (i.e., 2 years or less) means that the herd replacement rate is relatively high, with frequent introductions of susceptible animals (either homebred gilts or purchased sows) into the herd population. If replacement sows are sourced from outside the farm, this process carries with it an increased risk of introduction of SIV into a herd (10). The findings reported here are in broad agreement with those of previous reports (17, 35), indicating that absence of an “all in, all out” management system and increased herd replacement rate were associated with an increased risk of SIV seropositivity in intensively managed swine herds in France and Spain.

If a farm was located within 1 km of a commercial poultry farm, the odds of the herd being SIV-seropositive was increased. The districts that were included in this study were in an area of Java where the density of commercial poultry farms is relatively high and where avian influenza H5N1 is endemic (36), which implies that swine farms in the same area are likely to be continuously exposed to avian influenza virus. We used ELISA to detect influenza A nucleoprotein antibodies, and it is possible that seropositivity in individual pigs could have been due to a cross-reaction between antibodies induced by influenza A subtype viruses from pigs (swine influenza) and those from poultry (avian influenza). In addition, SIVs are known to contain combinations of genes originating from humans and poultry (37), and some avian influenza viruses (non-human type has) can transmit directly and even continuously circulate in pigs (6). It is known that HPAI H5N8 virus particles can be detected in air sampled between 50 and 110 m from infected poultry farms (38), and influenza A viruses have been found in air samples between 1.5 and 2.1 km from poultry in Southern Minnesota and Northern Iowa (39). In Canada, it was found that pigs could be infected with avian H4N6 viruses [70]. Collectively, these findings support the hypothesis that avian influenza viruses can cross species and cause influenza infections in swine.

Purchasing replacement pigs only from pig collectors/traders was associated with an increased odd of SIV-seropositivity, consistent with the findings of a previous study that showed that this practice increased the risk of swine influenza H1N1 and H3N2 infection (19). In this area of Java, swine collectors source pigs for sale from numerous sources with varying levels of biosecurity, providing a biologically plausible explanation for our findings. Raising industry awareness of the role that collectors play as facilitators for pathogen transmission is important, with perhaps gains to be made by applying tighter controls on pig collectors who purchase pigs from farms located in poultry-dense areas.

With an area under the curve value of 0.78, we conclude that our final logistic regression model had moderate to good ability to discriminate between SIV-seropositive and SIV-seronegative herds (34). Our model was highly specific but had relatively poor sensitivity. This means that, when the model predicted that a herd was going to be SIV-seropositive, on 95% of occasions this prediction was correct. In contrast, there was a substantial proportion of herds (0.64; 95% CI = 0.56–0.70) that were truly SIV-seropositive that were not detected as such using the explanatory variables included in the final model (Table 1). These findings show that, while this study has been useful for identifying (or at least confirming) herd-level risk factors for SIV seropositivity, other risk factors remain. Detailed interviews with herd managers that had SIV-seropositive herds but were in the reference group for each of the risk factors listed in Table 1 would be the first step toward identifying additional herd-level SIV seropositivity risk factors.

## Conclusions

Our results show that the prevalence of SIV exposure in swine herds in this part of Indonesia is relatively high and that SIV-seropositive herds in Java were geographically dispersed. The presence of other animal species on farm, herds with a relatively high replacement rate, herds that were located in close proximity to poultry farms, and the routine practice of purchasing pigs only from a collector were all associated with an increased risk of the herd being SIV-seropositive.

## Data Availability Statement

The datasets generated for this study are available on request to Directorate General Livestock and Animal Health Services (DGLAHS), Ministry of Agriculture (MOA), Jakarta, Indonesia.

## Ethics Statement

Ethical approval was not required for this study according to national/local legislation because this was a retrospective study that evaluated national pig disease surveillance data (data from February 2016 to Novermber 2017) from Disease Investigation Center (DIC) Subang within the Directorate General of Animal Health and Directorate General of Livestock and Animal Health Services of the Ministry of Agriculture, Indonesia. No research on animals was undertaken, other than analysis of existing surveillance data.

## Author Contributions

VP and N: study design and concept. FZ, LS, and N: surveillance design and concept. FZ and TM: data management and lab testing. N, VP, MS, and CP: data analyses. N, VP, and FZ: drafting the manuscript. All authors: reviewed and finalized the manuscript.

## Conflict of Interest

The authors declare that the research was conducted in the absence of any commercial or financial relationships that could be construed as a potential conflict of interest.
