# Supplementary material for: Herd-Level Risk Factors for Swine Influenza (H1N1) Seropositivity in West Java and Banten Provinces of Indonesia (2016–2017)
Source: Front Vet Sci. 2020 Nov 11;7:544279. doi: 10.3389/fvets.2020.544279 (PMC7685993; doi:10.3389/fvets.2020.544279)
Supplement: Supplementary file 1 [file Table_1.docx]

**Table S1**. Univariable logistic regression analysis between swine influenza farm status and the 29 explanatory variables for swine influenza virus in West Java and Banten provinces, Indonesia, 2016-2017.

| **Independent Variable** | **Cases** | **Control** | ***P-value*** | **OR (95% CI)** |
| --- | --- | --- | --- | --- |
| Age of farm: |  |  |  |  |
| Old (31-51 years) | 10 | 48 | 0.15 | 2.18(0.78-6.08) |
| Medium (15-30 year) | 10 | 22 |  | 2.11 (0.95-5) |
| New (1-14 years) | 26 | 59 |  |  |
| Type of farm: |  |  |  |  |
| Commercial | 3 | 17 | 0.2 | 2.16(0.58-12.11) |
| Individual | 43 | 112 |  |  |
| Population category: |  |  |  |  |
| Large (122-759 tails) | 14 | 42 | 0.4 | 0.87(0.38-2) |
| Medium (61-121 tails) | 16 | 55 |  | 1.5(0.64-3.55) |
| Small (1-60 tails) | 16 | 32 |  |  |
| Type of farm base on pig age: |  |  |  |  |
| Adult | 5 | 12 | 0.9 | 0.84(0.29-2.80) |
| Mixed | 40 | 113 |  | 0.6(0.02-5.50) |
| Young | 1 | 4 |  |  |
| Type of commercial: |  |  |  |  |
| Breeding | 12 | 21 | 0.03 | 0.67(0.30-1.56) |
| Combination | 32 | 83 |  | 0.14(0.02-0.58) |
| Fattening | 2 | 25 |  |  |
| Length of time sows kept on farm: |  |  |  |  |
| Less than 2 years | 41 | 97 | 0.04 | 2.69(0.94-9.47) |
| More than 2 years | 5 | 32 |  |  |
| Farm organize: |  |  |  |  |
| Worker | 10 | 40 | 0.15 | 8(0.69-182.71) |
| Owner and worker | 1 | 2 |  | 1.54(0.71-3.5) |
| Owner | 34 | 88 |  |  |
| Personal protection equipment: |  |  |  |  |
| Yes | 42 | 117 | 0.9 | 1.07(0.30-4.83) |
| No | 4 | 12 |  |  |
| Farm access: |  |  |  |  |
| Everyone | 15 | 47 | 0.6 | 1.18(0.55-2.61) |
| Owner | 31 | 82 |  |  |
| Distance farm to residence: |  |  |  |  |
| Outside residence (5-10 KM) | 5 | 36 | 0.01 | 3.15(1.12-11.04) |
| In the resident area (less than 5KM) | 41 | 93 |  |  |
| Farm management: |  |  |  |  |
| Caging | 43 | 127 | 0.08 | 4.3(0.48-54.09) |
| Mixed | 3 | 2 |  |  |
| Cage distance to household: |  |  |  |  |
| More 100 m | 12 | 61 | 0.01 | 2.52(1.15-5.86) |
| Less 100 m | 34 | 68 |  |  |
| Type of building farm: |  |  |  |  |
| Concrete | 38 | 96 | 0.08 | 0.22(0.03-0.83) |
| Mixed | 2 | 22 |  | 1.37(0.44-3.39) |
| Wood | 6 | 11 |  |  |
| Type of cage: |  |  |  |  |
| Colony | 40 | 113 | 0.91 | 1.05(0.31-3.10) |
| Colony and battery | 6 | 16 |  |  |
| Presence of other animal species on farm: |  |  |  |  |
| Yes | 32 | 72 | 0.1 | 1.8(1.84-4.02) |
| No | 14 | 57 |  |  |
| Having poultry: |  |  |  |  |
| Yes | 14 | 44 | 0.68 | 0.84(0.21-2.46) |
| No | 32 | 85 |  |  |
| Type of feed: |  |  |  |  |
| Bran | 41 | 112 | 0.91 | 1.05(0.33-2.96) |
| Swill | 5 | 17 |  |  |
| Feed frequency: |  |  |  |  |
| 1x | 1 | 6 | 0.21 | 2.11(0.34-40.54) |
| 2x | 43 | 112 |  | 12(0.611-556.63) |
| 3x | 2 | 1 |  |  |
| Water source: |  |  |  |  |
| Ground water | 14 | 48 | 0.18 | 1.2(0.61-2.67) |
| Surface water | 29 | 79 |  | 5.14(0.78-42.09) |
| Tap water | 3 | 2 |  |  |
| Cleaning and disinfection activity: |  |  |  |  |
| Yes | 30 | 90 | 0.64 | 1.32(0.62-2.73) |
| Cleaning | 15 | 34 |  | 0.6(0.03-3.91) |
| No | 1 | 5 |  |  |
| Slaughter facility in the farm: |  |  |  |  |
| Yes | 42 | 118 | 0.97 | 1.02(0.22-3.69) |
| No | 4 | 11 |  |  |
| Waste management: |  |  |  |  |
| Manure | 41 | 102 | 0.27 | 0.71(0.10-3.08) |
| Other | 2 | 7 |  | 0.37(0.08-1.16) |
| Sewer | 3 | 20 |  |  |
| Existence poultry farm: |  |  |  |  |
| Yes | 34 | 63 | 0.003 | 2.9(1.34-6.84) |
| No | 12 | 66 |  |  |
| Distance to nearest poultry farm: |  |  |  |  |
| Less than 1 km | 31 | 61 | 0.01 | 2.29(1.08- 5.03) |
| More than 1 km | 15 | 68 |  |  |
| Present poultry in the farm: |  |  |  |  |
| Yes | 22 | 50 | 0.28 | 1.44(0.69-3.01) |
| No | 24 | 79 |  |  |
| Slaughter poultry in the farm: |  |  |  |  |
| Yes | 5 | 4 | 0.04 | 3.77(0.77-19.97) |
| No | 41 | 125 |  |  |
| Vaccination program: |  |  |  |  |
| Yes (for other disease) | 2 | 12 | 0.28 | 0.44(0.04-2.12) |
| No | 44 | 117 |  |  |
| Deworming program: |  |  |  |  |
| Yes | 42 | 119 | 0.83 | 0.88(0.23-4.06) |
| No | 4 | 10 |  |  |
| Replacement pigs purchased only from a collector: |  |  |  |  |
| Yes | 23 | 19 | 1.51-06 | 5.7(2.53-13.18) |
| No | 23 | 110 |  |  |
